# Paeonol Attenuates Methotrexate-Induced Cardiac Toxicity in Rats by Inhibiting Oxidative Stress and Suppressing TLR4-Induced NF-*κ*B Inflammatory Pathway

**DOI:** 10.1155/2020/8641026

**Published:** 2020-02-10

**Authors:** Abdulla Y. Al-Taher, Mohamed A. Morsy, Rehab A. Rifaai, Nagwa M. Zenhom, Seham A. Abdel-Gaber

**Affiliations:** ^1^Department of Physiology, Biochemistry and Pharmacology, College of Veterinary Medicine, King Faisal University, 31982 Al-Ahsa, Saudi Arabia; ^2^Department of Pharmaceutical Sciences, College of Clinical Pharmacy, King Faisal University, 31982 Al-Ahsa, Saudi Arabia; ^3^Department of Pharmacology, Faculty of Medicine, Minia University, 61511 El-Minia, Egypt; ^4^Department of Histology, Faculty of Medicine, Minia University, 61511 El-Minia, Egypt; ^5^Department of Biochemistry, Faculty of Medicine, Minia University, 61511 El-Minia, Egypt

## Abstract

Methotrexate (MTX) is a commonly used chemotherapeutic agent. Oxidative stress and inflammation have been proved in the development of MTX toxicity. Paeonol is a natural phenolic compound with various pharmacological activities including antioxidant and anti-inflammatory properties. The aim of the present study was to evaluate the protective effect of paeonol against MTX-induced cardiac toxicity in rats and to evaluate the various mechanisms that underlie this effect. Paeonol (100 mg/kg) was administered orally for 10 days. MTX cardiac toxicity was induced at the end of the fifth day of the experiment, with or without paeonol pretreatment. MTX-induced cardiac damage is evidenced by a distortion in the normal cardiac histological structure, with significant oxidative and nitrosative stress shown as a significant increase in NADPH oxidase-2, malondialdehyde, and nitric oxide levels along with a decrease in reduced glutathione concentration and superoxide dismutase activity compared to the control group. MTX-induced inflammatory effects are evidenced by the increased cardiac toll-like receptor 4 (TLR4) mRNA expression and protein level as well as increased cardiac tumor necrosis factor- (TNF-) *α* and interleukin- (IL-) 6 levels along with increased nuclear factor- (NF-) *κ*B/p65 immunostaining. MTX increased apoptosis as shown by the upregulation of cardiac caspase 3 immunostaining. Paeonol was able to correct the oxidative and nitrosative stress as well as the inflammatory and apoptotic parameters and restore the normal histological structure compared to MTX alone. In conclusion, paeonol has a protective effect against MTX-induced cardiac toxicity through inhibiting oxidative and nitrosative stress and suppressing the TLR4/NF-*κ*B/TNF-*α*/IL-6 inflammatory pathway, as well as causing an associated reduction in the proapoptotic marker, caspase 3.

## 1. Introduction

Methotrexate (MTX), a cytotoxic chemotherapeutic drug with folate antagonistic activity, is commonly used in the treatment of various types of malignancies such as lymphoma [1]. It is also used at a low dose in the treatment of several autoimmune diseases such as rheumatoid arthritis [2]. MTX primarily inhibits the dihydrofolate reductase enzyme and secondarily inhibits purines and pyrimidines necessary for DNA and RNA synthesis; as a consequence, both contribute to the cytotoxic effects of MTX [3]. The cytotoxic effects of MTX are not restricted to tumor cells but also extend to affect vital organs [4] including the heart [5], where the inhibitory action of MTX on glyoxalase and antioxidant systems may also contribute to the toxicity of the drug [6]. Several agents have been used, with various degrees of success, to prevent MTX cardiac toxicity [7, 8].

Paeonol, a phenolic compound isolated from *Paeonia suffruticosa*, has been shown to have a wide range of pharmacological activities including antioxidant and anti-inflammatory properties [9] that may be beneficial in various diseases such as diabetes [10], cancer [11], gastric ulcer [12], liver fibrosis [13], and myocardial infarction [14]. Paeonol has been proven to have a protection against antineoplastic-induced toxicities such as cisplatin-induced nephrotoxicity [15] and epirubicin-induced cardiac toxicity [16]; however, to date, this role has not been investigated in MTX-induced cardiac toxicity.

Toll-like receptor 4 (TLR4), an important member of the TLR family, activates nuclear factor- (NF-) *κ*B and enhances production of inflammatory mediators such as tumor necrosis factor- (TNF-) *α* and interleukin- (IL-) 6 [17]. Thus, as TLR4 is expressed in cardiomyocytes [18], they are implicated in the development and progression of different myocardial inflammations such as myocarditis [19], myocardial infarction [20], ischemia-reperfusion injury [21], and heart failure [22]. In addition, TLR4 antagonists improved various cardiovascular diseases [23, 24]. Therefore, the aim of the current study was to evaluate the protective effect of paeonol in MTX-induced cardiac toxicity in rats and to evaluate the various mechanisms that underlie this effect.

## 2. Materials and Methods

### 2.1. Chemicals and Antibodies

Paeonol (2′-hydroxy-4′-methoxyacetophenone; molecular weight: 166.17; 99% pure powder) was purchased from Sigma-Aldrich Corp. (St. Louis, MO, USA), MTX was purchased from Minapharm Pharmaceuticals (Cairo, Egypt), and ready-to-use NF-*κ*B/p65 and caspase 3 rabbit polyclonal antibodies were purchased from Thermo Fisher Scientific Inc. (Waltham, MA, USA). Rat TLR4, TNF-*α*, and IL-6 enzyme-linked immunosorbent assay (ELISA) kits were purchased from Elabscience (Houston, TX, USA). All other chemicals were obtained from commercial sources.

### 2.2. Experimental Design

Male Wistar rats weighing 180–210 g were used after 1 week for proper acclimatization to the animal house conditions (12 h lighting cycle and 25 ± 2°C temperature). Rats were housed three rats per cage, and they had free access to commercial laboratory chow and tap water throughout the experiment. The animal ethical standards were in accordance with EU directive 2010/63/EU. Animals were divided into three groups (*n* = 6 each): (a) the control nontreated group, (b) the MTX-treated group that received a single intraperitoneal dose of 20 mg/kg MTX [25] at the end of the fifth day of the experiment, and (c) the MTX/paeonol-treated group treated by a single daily oral dose of 100 mg/kg paeonol suspended in carboxymethyl cellulose [26] for ten consecutive days and received MTX at the end of the fifth day of the experiment.

### 2.3. Sample Preparation

After 5 days of MTX injection, total body weights of rats were recorded, then rats were sacrificed and hearts were dissected out. Cardiac samples were homogenized in 10% *w*/*v* ice-cold phosphate buffer (0.01 M, pH 7.4). The homogenate was centrifuged at 3000 rpm for 20 min, and the supernatant was used for the estimation of NADPH oxidase- (NOX-) 2, malondialdehyde (MDA), nitric oxide (NO), reduced glutathione (GSH), TLR4, TNF-*α*, and IL-6 levels along with superoxide dismutase (SOD) activity. Ventricular samples were kept in 10% neutral-buffered formalin for histopathological and immunohistochemical examinations. Cardiac tissue samples were kept at -80°C for the determination of the TLR4 gene using real-time polymerase chain reaction (PCR).

### 2.4. Histopathological Evaluation of the Cardiac Damage

Cardiac specimens were fixed in 10% neutral-buffered formalin, embedded in paraffin, sectioned at 5 *μ*m thickness, and stained with hematoxylin and eosin. A modified scoring system was done according to Li et al. [14] and Moreno Júnior et al. [27]. Ten fields of vision for each group were selected. Histological changes such as necrosis of myofibers, hemorrhage, and inflammatory cell infiltration were scored on a 4-point scale: (-) negative, indicating no necrotic foci; (+) mild, indicating one or two small foci of myocardial necrosis, with scattered areas of hemorrhage and a slight degree of inflammatory process; (++) moderate, indicating more than two areas of small foci of myocardial necrosis, with a few areas of hemorrhage and a diffuse inflammatory process; and (+++) severe damage, indicating confluent foci of myocardial necrosis, with many areas of hemorrhage and massive areas of inflammation.

### 2.5. Evaluation of Cardiac Oxidative and Nitrosative Stress Markers

Oxidative stress markers were determined in a cardiac homogenate where NOX-2, MDA, NO, and GSH levels as well as SOD activity were evaluated. For quantitative determination of NOX-2, the Rat NOX-2 ELISA Kit (Bioassay Technology Laboratory, Shanghai, China) was used according to the manufacturer's instructions. The MDA level was determined by the assessment of thiobarbituric acid reacting substance through spectrophotometric measurement of color at 535 nm, and the results were expressed as nmol/g tissue [28]. NO determination, an indicator of nitrosative stress, was done chemically by the Griess method, which estimates the stable oxidation end products of NO, namely, nitrite (NO_2_^−^) and nitrate (NO_3_^−^), as indicators of NO production. The principle of the assay is the reduction of NO_3_^−^ to NO_2_^−^ by copperized cadmium granules, followed by color development with a Griess reagent in acidic medium that is measured spectrophotometrically at 540 nm; the results are expressed as nmol/g tissue [29]. The GSH determination method is based on the reduction of Ellman's reagent by thiol groups of GSH to produce 5-thio-2-nitrobenzoic acid that has a yellow color, which was measured spectrophotometrically at 412 nm and expressed as nmol/g tissue [30]. Cardiac SOD activity was measured colormetrically at 420 nm by the method that is based on the inhibition of pyrogallol autoxidation by SOD, and the results were expressed as U/g tissue [31].

### 2.6. Determination of Cardiac TLR4

Total RNA was extracted from a cardiac tissue homogenate using the RiboZol Reagent (AMRESCO, Solon, OH, USA) according to the manufacturer's instructions. Real-time PCR was performed with 50 ng of RNA template per reaction using the SensiFAST™ SYBR® Hi-ROX One-Step Kit (Meridian Bioscience Inc./Bioline; Memphis, TN, USA) in 25 *μ*l reaction volume containing 70 nM of specific primers in the Applied Biosystems 7500 Fast Real-Time PCR System (Foster City, CA, USA). Sequences of the primers were as follows: TLR4 forward primer, 5′-AATCCCTGCATAGAGGTACTTCCTAAT-3′ and reverse primer, 5′-CTCAGATCTAGGTTCTTGGTTGAATAAG-3′ [32] and glyceraldehyde-3-phosphate dehydrogenase (GAPDH) forward primer, 5′-GTCGGTGTGAACGGATTTG-3′ and reverse primer, 5′-CTTGCCGTGGGTAGAGTCAT-3′ [33]. The SYBR green data were analyzed with a relative quantification to GAPDH as a reference gene. The relative expression levels of the TLR4 gene were calculated according to VanGuilder et al. [34]. They were scaled relative to controls where control samples were set at a value of 1. For quantitative determination of cardiac TLR4 protein levels, a rat TLR4 ELISA kit was used according to the manufacturer's instructions.

### 2.7. Immunohistochemical Expression of NF-*κ*B/p65 and Caspase 3 in Cardiac Tissue

Sections were deparaffinized, hydrated, and washed with phosphate-buffered saline (PBS). Endogenous peroxidase activity was blocked using H_2_O_2_. Antigen retrieval was done by boiling sections in citrate buffer. Sections were incubated with NF-*κ*B/p65 and caspase 3 primary antibodies and preserved overnight at 4°C. Then, sections were washed with PBS and treated with goat anti-rabbit IgG secondary antibody. Sections were incubated with the streptavidin-biotin complex, and the color was developed using diaminobenzidine and counterstained with hematoxylin. For semiquantitative analysis, the mean number of immunopositive cells was detected in ten high-power fields of the sections in all groups.

### 2.8. Determination of Cardiac Inflammatory Cytokines

The inflammatory cytokines TNF-*α* and IL-6 were determined in a cardiac homogenate using rat TNF-*α* and IL-6 ELISA kits according to the manufacturer's instructions.

### 2.9. Statistical Analysis

GraphPad Prism software was used for statistical analysis (version 6.01 for Windows, San Diego, CA, USA). Results were expressed as means ± SEM. One-way analysis of variance (ANOVA) followed by Tukey's postanalysis test was used to analyze the results for statistically significant difference. Differences with *p* value < 0.05 were considered significant.

## 3. Results

### 3.1. Effect of Paeonol Treatment on Cardiac Histopathology

**Figure 1:**
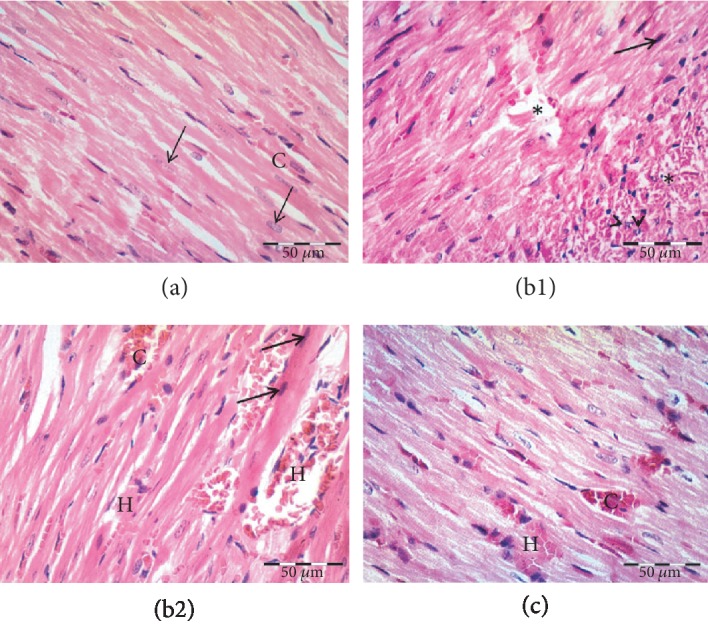
Effect of paeonol on histopathological changes in methotrexate- (MTX-) induced cardiac toxicity in rats. (a) Control group showing branched striated cardiac muscle fibers with acidophilic cytoplasm and central, vesicular, and oval nuclei (arrows). Notice the blood capillaries between the muscle fibers (C). (b1 and b2) MTX-treated group showing area of widely separated and fragmented necrotic cardiac muscle fibers (^∗^), congestion (C), and hemorrhage (H). Notice apoptotic muscle fibers, some with hyperacidophilic cytoplasm and pyknotic nuclei (arrows), others showing fragmented nuclei (arrowheads). (c) Paeonol+MTX group showing cardiac muscle fibers that appear more or less normal. Notice dilated congested blood capillaries (C) between the cardiac muscle fibers and area of hemorrhage (H).

Light microscopic examination of the control group showed branched cardiac muscle fibers with cross-striations, acidophilic cytoplasm, and central vesicular oval nuclei. Blood capillaries were noticed within the connective tissue stroma between the muscle fibers (Figure 1(a), Table 1). Sections of the MTX-treated group showed fragmented necrotic muscle fibers that appeared widely separated. Some fibers assumed apoptotic morphology with hyperacidophilic cytoplasm, nuclear pyknosis, and nuclear fragmentation. Many areas of hemorrhage and congestion were noticed (Figures 1(b1) and 1(b2), Table 1). In the paeonol+MTX group, most of the cardiac muscle fibers retained their normal appearance. Few scattered areas showed degenerated fibers. Many blood capillaries appeared dilated and congested (Figure 1(c), Table 1). Our preliminary experiments showed that paeonol alone had no effect on cardiac histopathology and oxidative stress markers (data are not shown).

### 3.2. Effect of Paeonol on Cardiac Oxidative and Nitrosative Stress Markers

In the MTX group, there was an increase in cardiac NOX-2, MDA, and NO levels, while there was a decrease in the level of GSH and in the activity of SOD in comparison to the normal control rats. Oral treatment with paeonol before MTX challenge significantly decreased the NOX-2, MDA, and NO levels and increased the level of GSH and the activity of SOD in comparison with the MTX group (Table 2).

### 3.3. Effect of Paeonol Treatment on Cardiac TLR4 mRNA Expression and Protein Level

**Figure 2:**
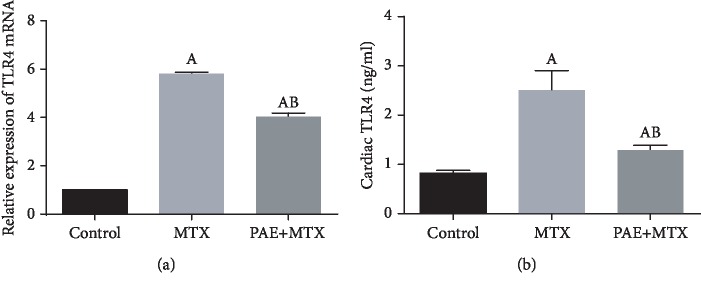
Effect of paeonol (PAE) on cardiac Toll-like receptor 4 (TLR4) mRNA expression (a) and protein level (b) in methotrexate- (MTX-) induced cardiac toxicity in rats. All parameters are expressed as means ± SEM. “A” means significantly different from the control group and “B” means significantly different from the MTX-treated group, at *p* < 0.05.

MTX-treated rats showed a significant increase in TLR4 mRNA expression in comparison to normal control rats. Paeonol pretreatment significantly decreased the TLR4 mRNA expression; however, it was still significantly higher than the normal control rats (Figure 2(a)). The same pattern was seen with the TLR4 protein level (Figure 2(b)).

### 3.4. Effect of Paeonol Treatment on Cardiac NF-*κ*B/p65 Immunostaining

**Figure 3:**
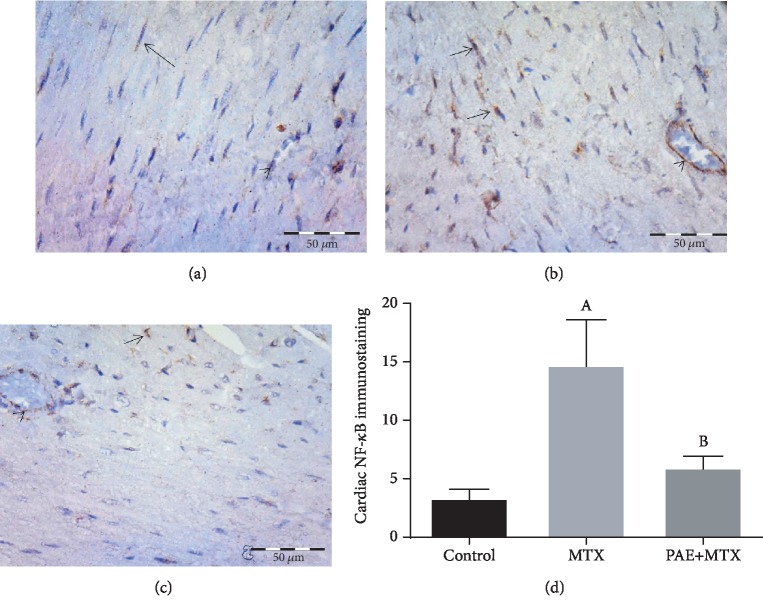
Effect of paeonol (PAE) on cardiac nuclear factor- (NF-) *κ*B/p65 immunostaining in methotrexate- (MTX-) induced cardiac toxicity in rats. (a) Control group showing positive expression in the cytoplasm of scattered cells of the endomysium (arrow). Notice negative expression in the endothelium (arrowhead). (b) MTX-treated group showing positive cytoplasmic and nuclear expression in many cells (arrows) and high expression in the endothelium (arrowhead). (c) PAE+MTX group showing positive expression in scattered cells (arrow) and low expression in the endothelium (arrowhead). (d) Semiquantitative analysis of the results. Values represent means ± SEM. “A” means significant difference compared to the control group and “B” means significant difference compared to the MTX-treated group, at *p* < 0.05.

Immunohistochemical staining of NF-*κ*B/p65 in the control group showed positive expression in the cytoplasm of scattered cells of the endomysium. The endothelium of the blood vessels showed either negative or low cytoplasmic expression (Figure 3(a)). However, the MTX-treated group showed positive cytoplasmic and nuclear expression in many cells. The endothelium of the blood vessels also showed high expression (Figure 3(b)). On the other hand, the paeonol+MTX group showed positive expression in a few scattered cells and low expression in the endothelium (Figure 3(c)). Semiquantitative analysis showed a significant increase (*p* < 0.05) in the mean number of the NF-*κ*B immunopositive cells in the MTX group compared to the control group. On the other hand, there was a significant decrease (*p* < 0.05) in the mean number of the immunopositive cells in the paeonol+MTX group compared to the MTX group (Figure 3(d)).

### 3.5. Effect of Paeonol Treatment on Cardiac Inflammatory Cytokines

**Figure 4:**
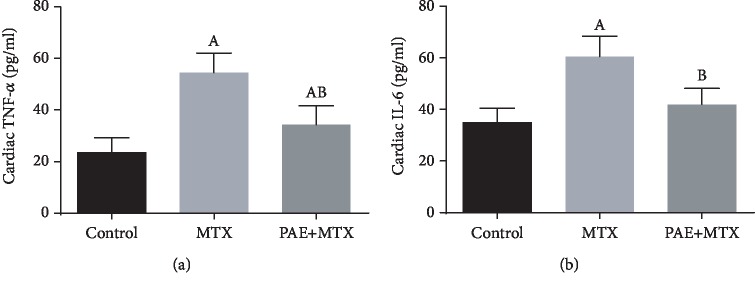
Effect of paeonol (PAE) on cardiac tumor necrosis factor- (TNF-) *α* (a) and interleukin- (IL-) 6 (b) levels in methotrexate- (MTX-) induced cardiac toxicity in rats. Values represent means ± SEM. “A” means significant difference compared to the control group and “B” means significant difference compared to the MTX-treated group, at *p* < 0.05.

MTX-intoxicated rats showed a significant increase in TNF-*α* and IL-6 levels in comparison with the control group. Paeonol-treated rats showed a significant decrease in these inflammatory cytokines compared to MTX-challenged rats (Figures 4(a) and 4(b)).

### 3.6. Effect of Paeonol Treatment on Cardiac Caspase 3 Immunostaining

**Figure 5:**
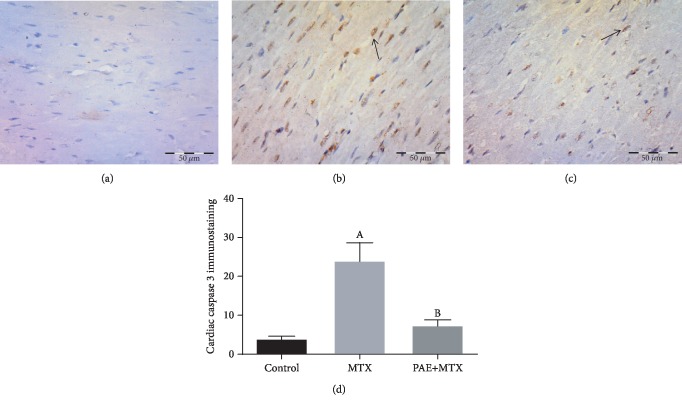
Effect of paeonol (PAE) on cardiac caspase 3 immunostaining in methotrexate- (MTX-) induced cardiac toxicity in rats. (a) Control group showing minimal expression. (b) MTX-treated group showing positive cytoplasmic and nuclear expression (arrow). (c) PAE+MTX group showing positive expression in scattered cells (arrow). (d) Semiquantitative analysis of the results. Values represent means ± SEM. “A” means significant difference compared to the control group and “B” means significant difference compared to the MTX-treated group, at *p* < 0.05.

Immunohistochemical staining of caspase 3 revealed minimal expression in control animals (Figure 5(a)). The MTX-treated group showed high cytoplasmic and nuclear expression in many cells (Figure 5(b)). Paeonol+MTX animals had a positive expression only in scattered cells (Figure 5(c)). Semiquantitative analysis showed a significant increase (*p* < 0.05) in the mean number of the caspase 3 immunopositive cells in the MTX group compared to the control group. On the other hand, there was a significant decrease (*p* < 0.05) in the mean number of the immunopositive cells in the paeonol+MTX group compared to the MTX group (Figure 5(d)).

## 4. Discussion

Cardiac toxicity after large doses of MTX was previously reported in the form of arrhythmias, hypotension, and cardiac arrest [5, 35], which necessitates multiple studies to evaluate agents with cardiac protective activities against the MTX-induced damage [7, 8]. The current results indicated that MTX treatment resulted in cardiac tissue damage manifested by histopathological changes of cardiac tissues. These results are in accordance with the findings of Tousson et al. [7] who reported that MTX treatment was associated with many histopathological abnormalities in rat cardiac tissues. On the other hand, in the present study, the alleviation of histopathologically examined MTX-induced cardiac tissue damage by paeonol is in line with the results of Wu et al. [16], who found that epirubicin-induced cardiac injuries were characterized by cytoplasmic vacuolization and interstitial hemorrhage; however, paeonol noticeably decreased this severe damage. Moreover, paeonol improved isoproterenol-induced myocardial histological changes such as necrotic changes in myofibrils with intense infiltration of neutrophil granulocytes and interstitial edema in rats [14].

MTX-induced oxidative and nitrosative stress plays an important role in the development and progression of MTX multiorgan toxicity including the heart [4, 7, 8]. MTX is known to elevate reactive oxygen species (ROS) by the elevation of homocysteine that is rapidly autooxidized forming ROS [36], reduction of NADPH that is utilized by glutathione reductase to retain the reduced state of glutathione that protects against ROS [37], and activation of NOX that generates ROS [38]. In the present study, MTX-induced oxidative cardiac tissue damage with disturbed oxidant/antioxidant balance is expressed as a significant increase in cardiac NOX-2 and MDA levels with a concurrent decrease in cardiac GSH level and SOD activity. In addition, MTX significantly increased the nitrosative stress marker NO level. These results are in accordance with the findings of Abdel-Daim et al. [8] who reported that MTX treatment was associated with a significant increase in MDA and NO levels together with a significant decrease in the GSH level and SOD activity.

The results of the present study have shown that paeonol prevents MTX-induced NOX-2 increase. In agreement with these results, paeonol decreased NOX-2 in rat primary microglia [39] and human primary chondrocytes [40]. In the current study, the ability of paeonol to significantly improve cardiac GSH level and SOD activity along with a decrease in MDA level is consistent with the finding of Li et al. [41] who reported that paeonol significantly increased cardiac GSH level and SOD activity besides decreasing MDA level in isoproterenol-induced myocardial injury in rats. NOX isoforms are a principal source of cardiac ROS and are associated with a wide range of cardiovascular diseases such as hypertension, atherosclerosis, heart failure, and cardiac arrhythmias [42]. GSH, a tripeptide nonenzymatic antioxidant, performs an essential role in antioxidant protection directly via scavenging ROS and indirectly via working as a cofactor of antioxidant enzymes [43]. SOD, an important first line antioxidant enzyme, catalyzes the dismutation of the superoxide radical into either molecular oxygen or hydrogen peroxide [44]. The inhibitory effect of paeonol on lipid peroxidation, assessed as MDA, could be secondary to its antioxidant activity and/or its repressing effect on inducible NO synthase- (iNOS-) mediated NO bioavailability [45, 46].

In harmony with the present study, several previous studies [47, 48] noted similar findings regarding the ability of paeonol to decrease the NO level. NO, a short-lived gasotransmitter, is known as a mediator and regulator of inflammatory responses. High levels of iNOS-derived NO are produced in response to inflammatory stimuli and mediate proinflammatory and destructive effects. These effects are mediated indirectly by a very quick interaction of NO with reactive oxygen species, which are synthesized by activated inflammatory cells resulting in the formation of the cytotoxic reactive nitrogen species peroxynitrite [49]. In addition, NO can activate NF-*κ*B, a transcription factor that controls many genes involved in inflammation [50]. NF-*κ*B is constitutively bound to inhibitor of *κ*B (I*κ*B) molecules, which confine its localization to the cytosol. After engagement of TLRs, signaling through MyD88 results in the phosphorylation of I*κ*B kinase. Phosphorylation of serine residues on I*κ*B promotes its degradation, thereby freeing NF-*κ*B to enter the nucleus and activate the transcription of target inflammatory genes. NF-*κ*B-regulated genes direct the differentiation of distinct immune cell types as macrophages. Differentiated macrophages produce different cytokines as TNF-*α* and IL-6 [51].

The present study showed that the MTX-induced cardiac inflammatory response (NF-*κ*B/TNF-*α*/IL-6) can be evidence of the increased cardiac TLR4 mRNA expression and protein level. These results are in accord with previous studies that demonstrated the MTX-induced increase in TLR4 and TNF-*α* mRNA [52] and NF-*κ*B/p65 immunostaining and TNF-*α* protein level [53] in the rat intestine. Moreover, Famurewa et al. [54] reported that MTX increased the IL-6 level in MTX-induced oxidative stress and inflammation in rats. On the other hand, in agreement with the current study, paeonol decreased the TLR4/NF-*κ*B/TNF-*α*/IL-6 inflammatory signaling pathway in lipopolysaccharide-induced acute lung injury [55] and IL-1*β*-induced human fibroblast-like synoviocyte rheumatoid arthritis [56]. Stimulation of TLR4 produces the activation of NF-*κ*B, which induces the generation of proinflammatory cytokines including TNF-*α* and IL-6 [17]. TLR4 also activates NOX to produce ROS [57]. In addition, inflammation and oxidative stress are interrelated as oxidative stress is involved in the activation of NF-*κ*B and the latter can induce oxidative stress [58], so it is difficult to predict a specific cause/effect relationship between them.

Caspase 3, a key enzyme in apoptosis, is activated in apoptotic cells through both extrinsic and intrinsic pathways [59]. The MTX-induced increase of caspase 3 has been reported in multiorgan toxicity [4, 25, 53, 60, 61]. Our results showed a significant increase in caspase 3 expression in response to MTX treatment. NF-*κ*B/p65 activation is required for endoplasmic reticulum stress-mediated apoptosis in cardiomyocytes [62]. Alternatively, stimulation of cardiomyocytes with ROS causes apoptosis [63]. Accordingly, in the current study, the effect of paeonol on caspase 3 expression, which concurs with various previous studies [12, 16], appears to be secondary to its antioxidant and anti-inflammatory activities. Remarkably, paeonol increased apoptosis and enhanced the cytotoxic effects of various anticancer drugs, such as doxorubicin [64], paclitaxel [65], and epirubicin [66]. Moreover, paeonol alone had its own cytotoxic effect [67, 68].

## 5. Conclusion

In summary, paeonol attenuates MTX-induced cardiac toxicity in rats partly through ameliorating oxidative stress by decreasing the NOX-2 level and preserving the level of GSH and activity of SOD as well as inhibiting the expression of the TLR4/NF-*κ*B/TNF-*α*/IL-6 inflammatory pathway. This was associated with a decrease in the level of the proapoptotic marker, caspase 3.

## Figures and Tables

**Table 1:** Effect of paeonol on the severity of histopathological lesions in methotrexate- (MTX-) induced cardiac toxicity in rats.

Groups	Myocardial necrosis	Hemorrhage	Inflammatory cell infiltration
Control	−	−	−
MTX	+++	++	+
Paeonol+MTX	+	+	+

Score level (−) is considered normal. Scores (+), (++), and (+++) are mild, moderate, and severe levels.

**Table 2:** Effect of paeonol on NADPH oxidase- (NOX-) 2, malondialdehyde (MDA), nitric oxide (NO), and reduced glutathione (GSH) levels along with superoxide dismutase (SOD) activity in methotrexate- (MTX-) induced cardiac toxicity in rats.

Groups	NOX-2 (ng/ml)	MDA (nmol/g tissue)	NO (nmol/g tissue)	GSH (nmol/g tissue)	SOD (U/g tissue)
Control	1.68 ± 0.10	27.20 ± 2.62	788.8 ± 73.8	1261 ± 13.4	5362 ± 350
MTX	6.01 ± 0.27^a^	56.77 ± 5.54^a^	3594 ± 244^a^	489.4 ± 41.7^a^	522.3 ± 47.4^a^
Paeonol+MTX	3.18 ± 0.13^a,b^	29.84 ± 0.43^b^	1780 ± 32.4^a,b^	751.6 ± 59.1^a,b^	4678 ± 445^b^

All parameters are expressed as means ± SEM of 6 observations. Significant difference is reported when *p* < 0.05. “a” means significant difference compared to the control group and “b” means significant difference compared to the MTX-treated group.

## Data Availability

The data used to support the findings of this study are available from the corresponding author upon request.
